# Systemic lupus erythematosus: from an adverse event of interferon administration to a disease with new treatment options

**DOI:** 10.48101/ujms.v130.13677

**Published:** 2025-12-17

**Authors:** Lars Rönnblom

**Affiliations:** Department of Medical Sciences, Rheumatology, Uppsala University, Uppsala, Sweden

**Keywords:** Systemic lupus erythematosus, interferon, etiology, pathogenesis, autoimmune, autoantibodies, immune complex, plasmacytoid dendritic cells, natural interferon producing cells, treatment, anifrolumab

## Abstract

Patients with systemic lupus erythematosus (SLE) display an increased expression of type I interferon (IFN)-regulated genes, a so-called IFN signature. This discovery was preceded by the observation in Uppsala that patients with malignant diseases treated with type I IFN occasionally developed autoimmune diseases, including SLE. The adverse event of IFN treatment was the start of an intensive search for the role of the type I IFN system in patients with spontaneously occurring SLE. A key finding by our group was the detection in patients with SLE of endogenous IFN-inducers that could activate plasmacytoid dendritic cells (pDC) to IFN production. Further studies revealed the mechanisms by which these cells are triggered to a continuous IFN synthesis. We could also identify a large number of risk genes for SLE and several molecules connected to type I IFN production and response. My group early on suggested the possibility that some of these molecules are suitable therapeutic targets in SLE, but also other IFN-driven diseases. Antibodies against the type I IFN receptor (anifrolumab) have recently shown efficacy in clinical trials for SLE, and anifrolumab is now approved as a treatment for this disease. Several other drugs targeting critical molecules in the IFN signaling pathways – including BCDA-2 (Blood Dendritic Cell Antigen 2), TLR7/8 (Toll-like receptor 7/8), and TYK2 (Tyrosine Kinase 2) – are currently in early clinical phases, potentially expanding therapeutic options for SLE. In this review, several important observations regarding the role of the type I IFN system in SLE and therapeutic implications are discussed.

**Figure:**
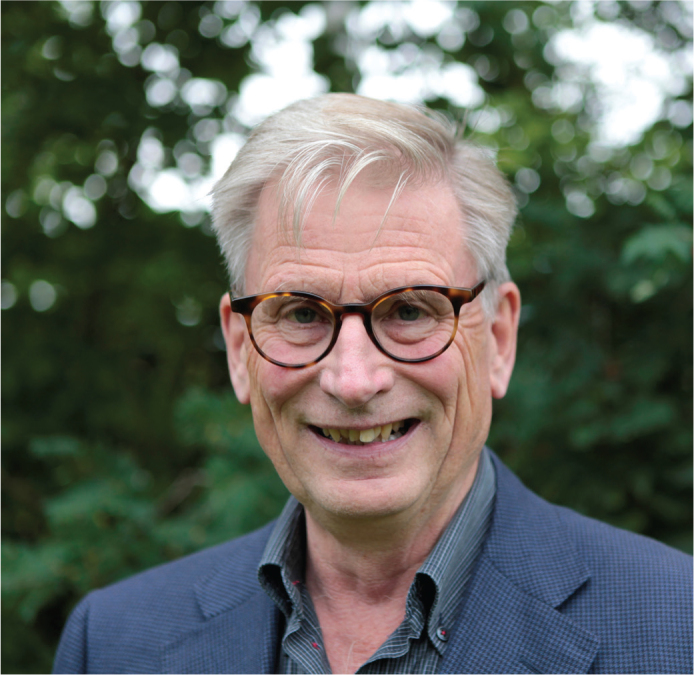


Professor Lars Rönnblom, winner of the Medical Faculty of Uppsala University Rudbeck Award 2025.

## Introduction

Systemic lupus erythematosus (SLE) is considered the prototype autoimmune disease because a large number of different autoantibodies (Abs) are produced, and most organs can be affected during the disease course. The clinical picture is very heterogeneous and spans from a relatively mild disease with skin and joint manifestations to a life-threatening disease with involvement of major organs, including the central nervous system (1). The different SLE subsets overlap, and a milder disease can progress over time to a more severe disease phenotype. The disease mainly affects women during their childbearing age and patients with SLE have a considerably reduced quality of life and increased mortality. For instance, the age-specific standard mortality rate in patients with SLE 19–39 years old is more than 12 times higher than in the general population (2).

In contrast to rheumatoid arthritis, few new therapies for SLE have been developed and approved during the last decades. There are several reasons for this, but the diversity of the clinical manifestations, the unpredictable disease course as well as the complexity of the immune aberrations in SLE, all contribute to a large number of treatment failures in clinical trials (3). These observations have raised the possibility that SLE in fact is not one disease, but rather many different disorders. The debate has not been completely settled yet, but as we learn more about the pathogenic mechanisms in SLE, a clear conclusion is that we need more precise personalized medicine to obtain remission in each patient with SLE (4).

An important contribution to the understanding of the pathogenesis of SLE was an early observation here in Uppsala that type I interferon (IFN) treatment of patients with malignant diseases could induce autoimmune diseases, including SLE (5, 6). This was an important clue to a possible role of the IFN system in the development of spontaneously occurring SLE, and the observation of the autoimmune adverse events after IFN treatment sparked intensive studies of SLE in Uppsala. Observations in patients with SLE also triggered a growing interest for the IFN system in patients with several other systemic autoimmune diseases, such as, for instance, Sjögren’s disease, myositis, and systemic sclerosis. In this review, several key findings with regard to the IFN system in SLE will be discussed, as well as some therapeutic consequences of this knowledge.

## Interferon production and treatment of patients with malignant diseases in Uppsala

IFN was originally described as an antiviral protein produced by virus-infected cells, with the capacity to interfere with viral replication in cells exposed to influenza virus (7). Subsequent studies revealed that IFN is not a single protein, but a large family of proteins that can be classified as type I, II, or III IFNs, of which type I IFN is the largest group of IFNs, with five classes (IFN-α, -β, -ε, -κ and ω). IFN-α can be further divided into 12 different subtypes, all with antiviral activity as well as strong immunostimulatory properties (8). As a consequence of the immunostimulatory effect by IFN, type I IFN was early on used not only for treatment of viral diseases, but also for patients with malignancies (9). In 1979, Professor Gunnar V Alm at the Biomedical Centre in Uppsala set up a production of type I IFN for clinical use. White blood cells from buffy coats were infected with Sendai virus for the induction of IFN synthesis, and the IFN was then purified and quality secured for use in patients. This human leukocyte IFN was a mixture of many different IFN-α subtypes and was used rather extensively for patients with malignant diseases at the Uppsala University Hospital. Beneficial effects were reported in some malignancies (10, 11), but early on several colleagues in Uppsala noted an increased occurrence of autoAbs and autoimmune disease during type I IFN treatment (5, 12, 13). As many as 19% of patients receiving long-term treatment with IFN-α eventually manifested an autoimmune disease (5).

A fascinating case was a young woman with a widespread malignant carcinoid tumor who developed arthritis, anemia, leukopenia, high titers of antinuclear antibodies (ANA) and anti-dsDNA antibodies during treatment with both human leukocyte IFN and recombinant IFN-α (6). IFN treatment was stopped due to the SLE syndrome and the symptoms subsided, although the ANA titer persisted. Surprisingly, the patient had a complete regression of the tumor, including all metastases, suggesting that the major target of the induced autoimmune reaction was tumor cells! The reason why this patient developed SLE is unclear, but she was probably predisposed to the disease, as SLE is a rare event during IFN treatment. She was typed as HLA A2,9; B5,7; DR2, but at this time gene sequencing was not available, so a more extensive genetic investigation was not possible. The clinical SLE manifestations disappeared as the IFN treatment was stopped, demonstrating that type I IFN was driving the autoimmune disease process.

## Early studies of the interferon system in patients with SLE

My PhD project at the IFN laboratory was to identify and characterize the blood cells that produced IFN after stimulation with viruses and other IFN inducers. An important finding in my thesis was the demonstration of a rare (1/1,000) cell type in peripheral blood with an extraordinary capacity to produce type I IFN (14, 15). In fact, one single IFN-producing cell could after stimulation produce 1 × 10^8^ IFN molecules in 24 h. These cells were designated ‘Natural IFN Producing Cells’ (NIPCs) as they didn’t express markers of previously well-characterized immune cells and were therefore termed in analogy with the Natural Killer cells (16). These cells are now usually denoted ‘plasmacytoid dendritic cells’ (pDCs), but there is an ongoing discussion as to whether the NIPCs comprise a separate subset within the large group of dendritic cells (17, 18). One support of this assumption is that only a subset of the pDCs population has the capacity to produce large amounts of IFN after stimulation (19).

After my clinical training in internal medicine and rheumatology, I decided, together with the rheumatology group, to explore the IFN system in patients with SLE whom I met at the rheumatology clinic. Such studies were logical also due to the fact that increased serum levels of IFN had been reported in samples from patients with SLE (20, 21), although it was difficult to determine if the IFN was type I or type II IFN from the beginning. Our initial studies of patients with SLE showed that the frequency of circulating NIPCs or pDCs in SLE is markedly reduced to 1/200,000 peripheral blood mononuclear cells (PBMC), but functional studies of SLE pDCs revealed that remaining single cells upon stimulation had a normal IFN-α producing capacity (22). This observation has been questioned, but the old Eli-spot technique used in these early studies is more sensitive than flow cytometry when looking for the function of extremely infrequent cells (23, 24). An unexpected, but very exciting, finding in this study was the induction of IFN production in PBMC from healthy individuals when exposed to sera from patients with SLE, see below.

Further studies suggested that the reason for the decreased number of circulating pDCs in SLE seems to be a migration of these cells to tissues, because an increased number of pDCs can readily be detected in skin (25, 26), lymph nodes (27), and renal tissue (28). These pDCs are activated in vivo and synthesize IFN-α, which indicates that these cells are at least partly responsible for the continuous IFN-α production seen in patients with SLE. Thus, aberrant pDC activation may be an important step in the process that eventually leads to SLE. However, it´s important to stress that also other cell types can be involved in IFN production (29). Among these are the keratinocytes, which can produce both IFN-κ (30) and IFN-λ (a type III IFN) (31), and monocytes, which have been implicated in the generation of the IFN signature (32). Neutrophils also have the capacity to produce type I IFN, and bone marrow-derived neutrophils in SLE patients produce IFN-α (33). Thus, several different cell types can contribute to the ongoing IFN production in SLE, and it´s conceivable that various IFN-producing cells may be important in different organs and in different patients during certain stages of the disease.

## Inducers of type I interferon production in SLE

In healthy individuals, type I IFN is produced in response to viral infections in order to limit viral replication and promote the antiviral adaptive immune response. During the first days after an infection, there is a prominent IFN peak, which rapidly declines as viruses are cleared. The ongoing IFN production in SLE therefore suggests the presence of endogenous IFN inducers or defects in the negative feedback system that controls the IFN response. An important finding was therefore the observation that sera from SLE patients have the capacity to trigger IFN production in cells from healthy individuals (34). After extensive studies we could show that immune complexes (IC) containing nucleic acids in SLE sera can activate pDCs (34, 35). Further studies revealed that such interferogenic ICs are internalized via the FcγRIIa expressed on pDCs, reach the endosome and stimulate the relevant Toll-like receptor (TLR) with subsequent activation of transcription factors and IFN-α production (36, 37). This mechanism for induction of type I IFN production has been demonstrated in vitro for both DNA- and RNA-containing ICs, and the nucleic acid-containing autoAgs in the interferogenic ICs were shown to be generated from apoptotic or necrotic cells (38), which is relevant given the increased apoptosis and reduced clearance of apoptotic cells in lupus (39, 40). Studies have shown that neutrophils undergoing so-called NETosis also have the capacity to provide interferogenic autoAgs (41, 42) demonstrating that several pathways can lead to pDC activation in SLE (29).

There is a correlation in SLE patients between serum IFN-α activity and the presence of autoAbs to RNA-binding proteins (43). Since some of these autoaAbs appear several years before the appearance of clinical overt lupus disease (44) and show cross-reactivity with viral epitopes (45), the initial trigger for the production of antibodies with IFN-α-inducing capacity could well be a viral infection. This scenario would connect viral infections with the generation of interferogenic ICs, which partly could explain the long-sought connection between infectious diseases and autoimmunity.

## Regulation of the type I interferon response

The continuous production of IFN in patients with SLE also raised the question of how the activation of pDCs is regulated, and whether some negative feedback mechanisms are defective in these patients. There is a large number of possibilities that could explain an inability to control an IFN response, from genetic defects to environmental factors that promote IFN production by blocking inhibitory signals. In early studies, we could show that the IFN response by pDC is regulated by reactive oxygen species (ROS) produced by monocytes and that this ROS production is reduced in patients with SLE (46). The defect in ROS production could later be shown to be caused by a single nucleotide polymorphism in the neutrophil cytosolic factor 1 (NCF1) gene leading to reduced oxidative burst (47). This gene polymorphism (NCF1-339 T allele) is associated to SLE and low-ROS patients also had increased serum IFN activity compared to patients with a normal-ROS NCF1-339 genotype (48). Recent studies have shown that this genotype directly can stimulate pDC generation and promote migration of these cells to organs affected in the SLE disease process (49).

A genetic defect in the complement component 1q (C1q) gene is a well-known risk factor for the development of SLE. We could show in vitro that C1q inhibits the IC-induced IFN-α production in pDC (50) and later report a patient with hereditary C1q deficiency characterized by both very high serum levels of IFN-α and high serum interferogenic activity (51). It’s now confirmed that hereditary C1q deficiency is connected to a strong IFN pathway activation and that the defect can be regarded as part of the interferonopathy spectrum of diseases (52).

The role of other cells than monocytes in the regulation of pDCs is of course of great interest, given the fact that most cells in the immune system are involved in the SLE disease process. Important observations were therefore that several cell types, once activated, can stimulate pDC to an increased IFN production. Thus, NK cells, B cells, and T cells all can enhance the IFN production when pDCs are exposed to nucleic acid containing ICs (53–55). The in vivo relevance of these findings remains to be established but suggests that in SLE there is extensive crosstalk between pDCs and different immune cells, which promote the ongoing IFN production and sustain the autoimmune process.

## The interferon system in the SLE disease process

Our observations in both IFN-treated patients and patients with SLE made that we put forward a hypothesis regarding an etiopathogenic role for the type I IFN system in SLE (56). An interesting part of this story is that it was very difficult from the beginning to get this opinion paper published, as an activation of the type I IFN system had not been demonstrated in any murine lupus models. The reason for this discrepancy between humans and mice was shown by Farrar to be due to a minisatellite insert in *STAT2* in all mice, causing a lack of STAT4 activation by IFN-α (57). Thus, there are major differences between humans and mice in the type I IFN system, which can explain the lack of type I IFN activation in the classical murine lupus models.

**Figure 1:**
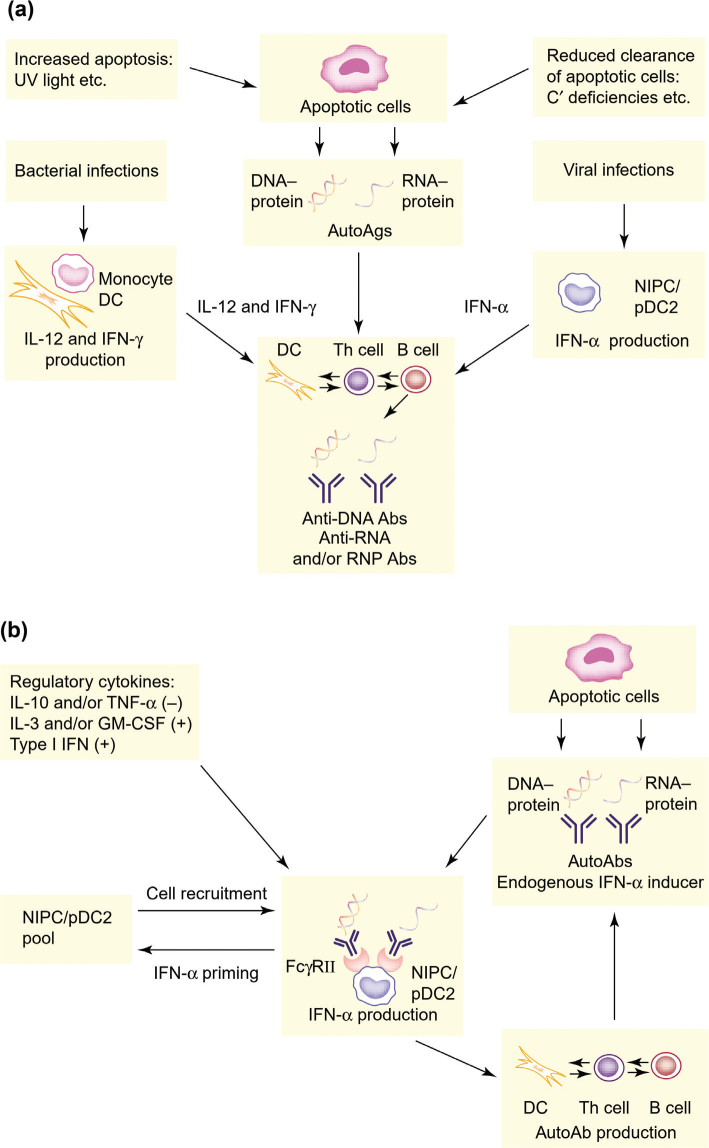
A possible role for the type I IFN system in the development of SLE. (a) In the first phase, protein autoantigens (autoAgs) associated to DNA or RNA are generated from apoptotic cells. These autoAgs become immunogenic in the presence of costimulatory cytokines produced in response to infections. (b) In the second phase, complexes between autoAbs and apoptosis-derived DNA- and/or RNA and associated proteins form, and can then act as endogenous IFN-α inducers. These inducers are generated for a long time and continuously activate the natural interferon producing cells (NIPCs)/plasmacytoid dendritic cells (pDC) to IFN-α synthesis. Produced IFN continues to stimulated the central autoimmune reaction by its effect on T and B cells as well as dendritic cells (DC) in a vicious circle mechanism. The figure is reproduced with permission from ‘Rönnblom L, Alm GV. An etiopathogenic role for the type I IFN system in SLE. Trends Immunol. 2001;22:427–31’.

Our novel disease model suggested that SLE develops in two distinct phases, and in Figure 1 the original figures from Trends in Immunology on our hypothesis regarding the etiopathogenesis of SLE are reproduced (56). Initially, auto- Abs against nucleic acid and associated proteins are generated during infections because of the costimulatory effects of type I IFNs and other cytokines (Figure 1a). In the second phase, ICs form and act as endogenous IFN-α inducers, which sustain the autoimmune process by prolonging the production of IFN-α (Figure 1b).

The first phase is dependent on the generation of immunogenic Ags from cells as a consequence of, for example, apoptosis. Such Ags are accessible to DCs, but are not normally immunogenic because of the production of the inhibitory cytokines transforming growth factor β (TGF-β) and IL-10, as well as the lack of costimulatory cytokines (58). However, when IFN-α is produced, for example, as a result of a viral infection, DCs can activate naive autoimmune T cells, which subsequently stimulate B cells to produce autoAbs directed against nucleic acid and associated proteins. This event is facilitated by the fact that the production of type I IFNs and Ag-presentation occur in similar, if not identical DCs. In addition to IFN-α, other costimulatory cytokines, such as IL-12 and IFN-γ, might also promote such production of autoAbs. Furthermore, the deficient clearance of apoptotic material and increased apoptosis could also contribute by providing more autoAgs.

This model has during the years been refined, and more components have been added to the picture, but the model in its general outline is still true and serves as a framework when pathogenic mechanisms and treatments for SLE are discussed (29, 59, 60).

## The genetics of SLE

The strong genetic impact on the risk of developing SLE has been obvious for decades, mainly manifested as a female: male incidence ratio of 10:1 in adults with SLE. Today there are more than 300 genetic loci associated with SLE, which have been possible to identify due to genome-wide association studies and whole-genome sequencing (61–63). The importance of gene variants in the IFN signaling pathway for SLE susceptibility was shown already in 2005, when we together with professor Ann-Christine Syvänen in a candidate gene study described the association between SLE and IFN regulatory factor 5 (*IRF5*) and tyrosine kinase 2 (*TYK2*) (64). IRF5 is involved in the production of IFN and TYK2 in the IFN response, but both have multiple functions in the immune system and can thus affect disease susceptibility by various mechanism, besides increased IFN signaling (65). The important role of many different immune pathways in SLE was shown when we found multiple molecular pathways associated to SLE in a study where we used targeted DNA sequencing of 1,832 immune related gene regions in patients and healthy controls (66). Two main independent pathways involved in SLE susceptibility were identified: T lymphocyte differentiation and innate immunity, characterized by HLA and IFN, respectively. Pathway polygenic risk score (PRS) analysis uncovered that SLE patients on average were positive for seven pathways, but some individuals had more than 20 affected pathways.

Important questions in this context are 1) what is the function of identified risk loci, 2) can this knowledge be used in a clinical setting, and 3) can the information be of value in drug development. During the last years, much data have been obtained that give some answers to these questions. One example is *STAT4* which early on was shown to harbor gene variants associated to multiple autoimmune diseases, including SLE (67, 68). *STAT4* has therefore often been referred to as a general autoimmunity risk gene. We could show how the major risk gene variant in intron 3 of *STAT4* affects the function of immune cells from patients with SLE in a cell-type specific and context-dependent manner (69). Activated CD8 T cells from SLE patients carrying the *STAT4* risk allele selectively displayed an augmented response to IL-12, which resulted in increased IFN-γ production, and an increased response to IFN-α. Interestingly, this increased response was not seen in cells from healthy individuals harboring the risk gene variant. However, when exposed to type I IFN, the cells switched to a ‘lupus phenotype’ with increased STAT4 phosphorylation (70). This latter observation could perhaps explain why a viral infection can induce an SLE syndrome in a healthy individual if the individual carries the STAT4 risk gene variant.

We could early on show that the *STAT4* risk gene is associated with increased risk for stroke, nephritis, and renal failure (71, 72). After we had explored the consequences of the STAT4 risk gene variant in T cells, we showed that there is a strong interaction in patients with SLE between the *STAT4* risk allele and smoking (73). Thus, patients with the risk gene variant have a clearly increased risk for both myocardial infarction and nephritis compared to smokers without the risk gene. A possible mechanism behind this association was the observation that levels of interleukin-12-induced phosphorylation of STAT4 in CD8+ T cells were higher in smokers than in non-smokers. The increased activation of CD8+ T cells from *STAT4* risk patients was efficiently blocked by Janus kinase inhibitors (JAKi) selective for TYK2 (TYK2i) or JAK2 (JAK2i), suggesting that this subset of patients may benefit from JAKi treatment.

## The IFN system and disease manifestations

**Figure 2:**
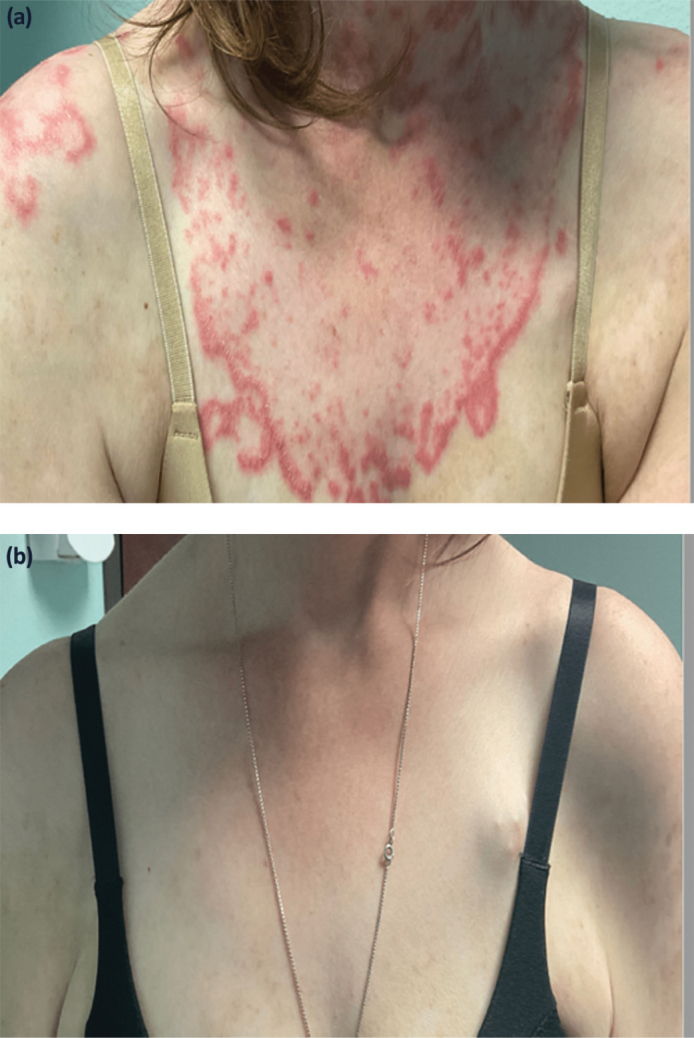
A 39 year old woman with SLE for 18 years and a treatment resistant rash (a), who was treated with anifrolumab and after 5 months she had a significant improvement (b). Case reported by Khan M, Khan F, Khan H, Saadeh C, Davey N. Role of anifrolumab in refractory cutaneous manifestations of lupus erythematosus: a case series and literature review. Cureus. 2023;15(5):e39553. doi: 10.7759/cureus.39553

Patients who are treated with type I IFN usually experience symptoms resembling a viral infection, i.e. fever, myalgia and fatigue. Similar symptoms are typical for patients with SLE, and there is an association between many signs and symptoms in patients with SLE and activation of the IFN system. In early studies we noted that serum IFN-α levels correlated with disease activity, fever, skin rash, anti-dsDNA levels and leucopenia (74). Skin manifestations are connected to infiltrating pDCs and expression of IFN-regulated genes (75), and a large number of studies have shown that targeting the IFN system in patients with SLE improves the skin manifestations (75–77) (see Figure 2). Today there is a general agreement that patients with a prominent IFN signature have a more severe and active disease (78), and infiltrating pDCs can be found in many organs. SLE nephritis is characterized by infiltrating pDCs as mentioned above, and an interesting observation is that a high IFN-response signature and fibrotic signature in tubular cells associates with failure to respond to conventional treatment (79). Thus, one possible reason for treatment failure in a subset of patients with SLE is that our traditional standard treatments don’t down-regulate the IFN system sufficiently to prevent organ inflammation and damage. This assumption is also supported by the strong connection between the STAT4 risk gene variant, enhanced CD8 T cell response and renal failure as discussed above.

## Targeting the IFN system

The standard treatment of patients with SLE includes hydroxychloroquine (HCQ), which prevents endosomal activation of TLRs and thereby blocks this pathway of inducing IFN gene transcription. It’s obvious, however, that HCQ doesn’t completely abolish the IFN gene expression, as many patients on HCQ treatment still display an IFN signature (78). Today a large number of drugs and treatment strategies have therefore been developed in order to inhibit IFN activation in SLE, and many of these are now in phase II/III clinical trials (80) (see Table 1). At the moment, anifrolumab is the only type I IFN inhibitor approved for treatment of patients with SLE, and in Sweden it’s used for moderate to severe and active autoantibody positive disease on standard of care. Anifrolumab has demonstrated efficacy in joint and skin disease, as well as reduction of flares and improved glucocorticoid tapering, with a tolerable safety profile (81). We early on suggested that pDC could be targeted in SLE and demonstrated that an anti-BDCA2 antibody could inhibit immune complex-mediated IFN production by pDC from lupus patients (82). Recent studies have shown efficacy of an anti-BDCA2 antibody (Litifilimab) for cutaneous lupus erythematosus (77) and this drug is now in several clinical trials for both cutaneous and systemic lupus. It’s also interesting to note that the gene products of two SLE susceptibility genes identified by us, namely TYK2 and IRF5, now are explored as therapeutic targets (Table 1). This illustrates the fact that a clear human genetic association to a proposed disease mechanism increases the success rate from clinical development to approval of a new drug for the disease targeting this pathway (83). Interleukin receptor 1-associated kinase 4 (IRAK4) is critical for the activation of pDC and blocking this signal transducer inhibits IC-induced type I and type III IFN production by pDC (84). At the moment one Phase IIa study targeting IRAK4 is ongoing in cutaneous lupus (85).

**Table 1:** Treatment options for inhibition of interferon (IFN) pathway activation.

Target	Drug	Mechanism	Comment
IFN inducers	RNase-Fc, RNase/DNase-Fc (RSLV-132)	Degrade RNA, DNA in IFN inducers	RNase-Fc improve IFN score in SjD
ILT7 CD123 BDCA2 on pDC	Daxdilimab CSL362 Litifilimab	Inhibit pDC function and IFN production	All show effect in early studies. Litifilimab now in phase III for SLE
TLR 7/8	E6742	Inhibit IFN production by blocking endosomal TLRs	Phase I/II studies show 90% Inhibition of IFN signature
IFN-α	Sifalimumab IFN-α kinoid	Bind to IFN-α protein Induce anti-IFN-α antibodies	Anti-IFN-α antibodies give a weak treatment effect
Type I IFN receptor	Anifrolumab	Bind to the type I IFN receptor and block IFN signaling	Approved for clinical use in SLE
TYK2 JAK/STAT	Deucravacitinib Baricitinib Tofacitinib Upadacitinib Filgotinib	Block IFN signaling downstream the type I IFN receptor	Response in phase II studies. Deucravacitinib and Upadacitinib are both in phase III studies
IRAK4	Several inhibitory small molecules (eg Edecesertib)	Block TLR and interleukin-receptor 1 pathways	Broad inhibition of proinflammatory pathways. Clinical studies ongoing
IRF5	KT-579, NS5 Degrade or inhibit IRF5	Broad effect on IFN and autoantibody production	Effect in murine models, Phase I planned 2026

SjD: Sjögrens Disease; ILT7: immunoglobulin-like transcript 7; CD123: IL-3 receptor α-chain; BDCA2: Blood Dendritic Cell Antigen 2; pDC: plasmacytoid Dendritic Cell; TYK2: Tyrosine Kinase 2; JAK/STAT: Janus kinase (JAK)-signal transducer and activator of transcription (STAT); IRAK4: interleukin-1 receptor-associated kinase 4; TLR: Toll-like receptor; IRF5; Interferon Regulatory Factor 5.

## Conclusions and further perspectives

A majority of patients with SLE display an IFN signature, despite a very heterogenous clinical picture. This is partly due to the fact that a large number of proinflammatory and regulatory pathways are activated in patients with SLE, besides the IFN system. In fact, the large family of IFNs regulates up to 10% of the genome and interacts with, and affects, most signaling pathways, making the IFN system one of the most complex and sophisticated biological networks in the body. Given these facts, it’s perhaps not surprising that our attempts to control and modulate the IFN system in patients with systemic autoimmune diseases so far have demonstrated moderate clinical success in large clinical trials. However, single patients have shown dramatic improvements in their disease after just a few doses of anifrolumab (Figure 2). This clinical experience underscores the need for personalized medicine, where each patient can receive individual treatment based on both the phenotype and endophenotype. To what extent comprehensive genetic and epigenetic information in combination with proteomics will let the clinicians reach this goal is at the moment unclear, but it is a realistic hope for the future.
